# Detecting Topological Defect Dark Matter Using Coherent Laser Ranging System

**DOI:** 10.1038/srep29519

**Published:** 2016-07-08

**Authors:** Wanpeng Yang, Jianxiao Leng, Shuangyou Zhang, Jianye Zhao

**Affiliations:** 1 School of Electronics Engineering and Computer Science, Peking University, Beijing, 100871, China

## Abstract

In the last few decades, optical frequency combs with high intensity, broad optical bandwidth, and directly traceable discrete wavelengths have triggered rapid developments in distance metrology. However, optical frequency combs to date have been limited to determine the absolute distance to an object (such as satellite missions). We propose a scheme for the detection of topological defect dark matter using a coherent laser ranging system composed of dual-combs and an optical clock via nongravitational signatures. The dark matter field, which comprises a defect, may interact with standard model particles, including quarks and photons, resulting in the alteration of their masses. Thus, a topological defect may function as a dielectric material with a distinctive frequency-depend index of refraction, which would cause the time delay of a periodic extraterrestrial or terrestrial light. When a topological defect passes through the Earth, the optical path of long-distance vacuum path is altered, this change in optical path can be detected through the coherent laser ranging system. Compared to continuous wavelength(cw) laser interferometry methods, dual-comb interferometry in our scheme excludes systematic misjudgement by measuring the absolute optical path length.

Many cosmological observations indicate that the matter-energy content of the universe is overwhelming dominated by cold dark matter (23%) and dark energy (73%)^1^. However, the question of dark matter (DM), including its identity, properties, and nongravitational interactions, remains one of the most important unsolved problems in contemporary physics. DM is a non-luminous, non-baryonic form of matter that interacts very weakly with itself and Standard Model(SM) matter. At early cosmological times, very light fields in the initial field configuration could lead to dark-matter via coherent oscillations around the minimum of their potential. Such dark matter configurations are entwined with spontaneous symmetry breakdown^2^. They are generally termed as topological defect dark matter (TDM) and can have various different types: 0D (corresponding to monopoles), 1D (strings), and 2D (domain walls).

The interaction of SM elementary particles (e.g. electrons and protons) with TDM particles may result in some instantaneous changes in their masses, which can lead to observable nongravitational signatures of DMs. Observable effects of TDMs can vary greatly, depending on their mass *m*_*a*_ from 10^−33^ to 10^5^ eV. The transverse size d of a defect cannot be predicted from existing theory in an ab initio manner, however, its characteristic transverse size is determined by the field Compton wavelength d, *d* ~ 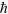/*m*_*a*_*c*.

Defects have primarily been sought for via their gravitational effects, including gravitational lensing and gravitational radiation, however, their results are usually less than satisfactory because they neither can confirm nor exclude the existence of cosmic defects. In more recent years, schemes for the detection of nongravitational effects induced by a defect passing directly though Earth have undergone astonishing developments, e.g, the pulsar glitch phenomenon method^3^, cw-laser interferometry methods^4^,^5^ and the global network of synchronised atomic magneto-meters^1^ or synchronised atomic clock method^6^. Based on the rapid development of high-precision measuring apparatus especially in laser physics (such as optical or atomic clock^7^,^8^, altra-stable fibre^9^, and high-sensitivity atomic magnetometer^10^), these novel DM searching schemes bring optimistic hope for direct detection of TDMs.

In the present work, we propose to use a high-precision coherent laser ranging system, which contains dual-comb and optical clock, to search for TDMs via nongravitational signatures. When a topological defect passes through Earth, the refractive index and the optical path length will change because of the coupling between photons and TDMs^3^, which could be detected through these dual-comb interferometry ranging systems^11^,^12^,^13^. In the past few decades, femtosecond comb technology has had a dramatic impact on the diverse fields of precision measurement and extreme nonlinear optical physics^14^,^15^. Many novel methods have been investigated to combine both high precision and long range measurement capability by exploiting the spectral properties of frequency combs^12^,^13^,^16^. Because of the easily achievable low frequency signal processing and the further extendible non-ambiguity range, dual-comb interferometry has attracted greater attention. Relying on analysis of the frequency spectrum of the interference signal to derive the ratio between the phase and the frequency, dual-comb interferometry^11^,^17^,^18^ is able to achieve a rapid absolute distance measurement with high resolution, which could be used for detecting topological defect dark matter.

## Results

**Figure 1:**
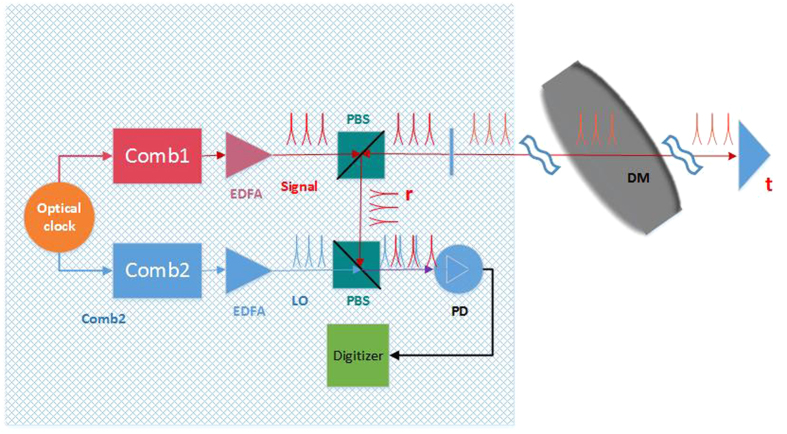
Schematic of the coherent laser ranging system that is used to search for TDMs. A high-repetition-rate śignalśource transmits pulses that are reflected from two partially reflecting planes: reflection plane and reference plane. The length of the optical path is measured as the time delay between two returned signal pulses.

Consider a topological defect dark matter passing directly through a long-distance laser interferometer(such as LIGO). An interaction occurs between the defect and photons, which increases the flight time frequired for the periodic pulses to make a round trip in the vacuum interferometer arms, in other words, it increases the optical path length of the arms. As previously mentioned, the pulsed nature of an optical comb is combined with the coherence of the carrier, allowing for a time-of-flight measurement simultaneously with an interferometric measurement based on the carrier phase^11^,^18^,^19^. A schematic of the TDM detection apparatus based on this characteristic nature of dual-comb is shown in Fig. 1. A pair of stabilised femtosecond laser frequency combs having pulse trains of slightly different repetition periods (*T*_*r*_ and *T*_*r*_ − Δ*T*_*r*_) are phase-locked to a common clock (details are in the **Method**). Comb 1 samples the distance path defined by reflection plane and reference plane. Comb 2 recovers the range information in an approach equivalent to linear optical sampling(that is, a heterodyne cross-correlation between the comb 1 and comb 2). The heterodyne detection can detect even weak return signals while retaining carrier phase information. Because of the slight repetition rate difference, The comb 2 pulses overlap the two returned comb 1 pulses every 
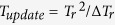
. The interference signal between the two combs pulses is detected in the lap position.

The relative spectral phase of the two returned comb 1 pulses reflected by two different planes can be calculated though D/A conversion and Fourier processing. The relative spectral phase between these two returned pulses is


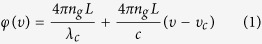


Where L is the length of the optical path, *λ*_*c*_ is the carrier wavelength and *υ*_*c*_ is the carrier frequency, *n*_*g*_ is the group refractive index at the carrier frequency. A simple linear fit 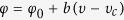 gives the coarse time-of-flight measurement value:


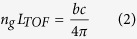


And the high-precision interferometric distance measurement value:


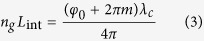


By substitution of the rudeness measurement value calculated from Eq. 2 into Eq. 3, the value of integer m can be confirmed. The measurement accuracy is mainly depend on Eq. 3, that is, the precision of *φ*_0_^11^. When a topological defect dark matter passes directly through the optical path, according to Eq. 3, *φ*_0_ is altered as the change of *n*_*g*_,


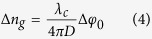


Where *D* = min {*L*, *d*} is the effective length of optical path in defect.Thus, TDMs directly passing through the Earth may be detected by measuring the shift of *φ*_0_ through coherent dual-comb interferometer method.

Furthermore, cw-laser interferometry methods^4^,^5^ are limited to observe whether the relative optical path length difference between two interferometer arms has changed, and are hardly able to determine the absolute variation. In contrast, our dual-comb interferometry ranging system can measure the absolute optical path length accurately. If the change of the optical path length is too large to exceed the estimation range caused by TDMs, our detection method will attribute it to system error, instead of the arrival of TDMs. Therefore, a part of the systematic misjudgement will be excluded by the absolute length measurement. In addition, a network composed of more than five detecting stations, which has been described in detail in our previous work^5^, is necessary to reduce the probability of misjudgement of TDMs.

## Discussion

There are many possibilities for the interactions of TDMs with the standard model particles. In the present discussion, we consider couplings with a quadratic dependence on the scalar DM field *ϕ*^3^


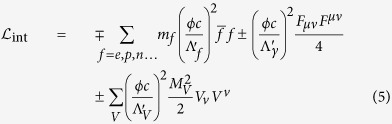


Where f is the fermion Dirac field and 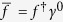, F is the electromagnetic field tensor and V are the components of the wave function of the corresponding massive vector boson. The high-energy scale 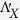 signifies the effective nature of TDMs, which is strongly constrained by astrophysical and atomic spectroscopy^20^. In this letter, we are only interested in the second term, which corresponds to the coupling between the dark matter field and photons. To necessitate the choice of gauge in flat spacetime: 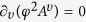, the coupling can be expressed as:


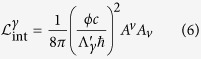


Which is similar to the coupling in Proca theory:


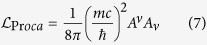


Comparing Eqs 6 and 7 shows the interaction of a photon with TDMs providing it with mass 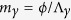, which will result in transient alteration of the index of refraction inside the defect^3^:


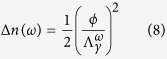


Where we denote 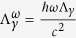 as the coupling strength between TDMs and photons with frequency *ω*. Thus, a defect may function as a cosmic dielectric material with a distinctive frequency-dependent index of refraction. According to Eqs 4 and 8, the *φ*_0_ shift due to the encounter with TDMs is given by:


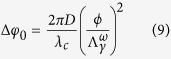


The energy density of TDM averaged over a large amount of defects is controlled by


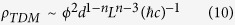


And the average time between ćlose encountersẃith defects is determined by the galactic velocity of such objects *υ*^5^:


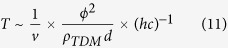


The maximum energy of density of the TDMs in the neighbourhood of the Solar System is constrained by the experimental estimated dark-matter energy density, 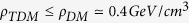. Thus,


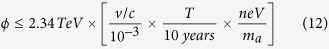


This constraint implies some flexible evolution of the TDMs and the possibility for them to build up their mass inside galaxies.

Finally, we estimate the sensitivity of detection system to effects stemming from topological defects. To make our discussion more specific, an example of a domain-wall (2D) network^21^ shown in Fig. 1 is considered. Considering a femtosecond optical frequency comb, the carrier-envelope phase, Φ_*ceo*_, the phase shift between the peak of the envelope and the closest peak of the carrier wave is connected to the comb offset 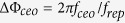. Therefore, the jitter of *f*_*ceo*_ and *f*_*rep*_ contribute to the uncertainty of *φ*_0_


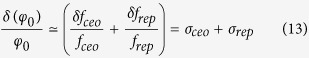


And the phase shift between two comb 2 pulses corresponding to the overlap of the two returned comb 1 pulses is given by


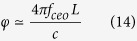


To determine the arrival of TDMs, the uncertainty of *φ*_0_ is must be satisfied *δ* (*φ*_0_) ≤ Δ*φ*_0_, thus,


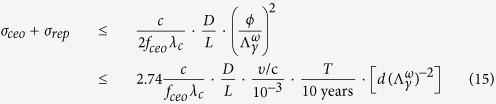


Where *σ*_*ceo*_ and *σ*_*rep*_ is the Allan variance of *f*_*ceo*_ and *f*_*rep*_, respectively; in the inequality the gravitational constraint from Eq. 12 is used. The unit of T, d, 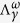 are taken to be year(yr), km and TeV, respectively.

**Figure 2:**
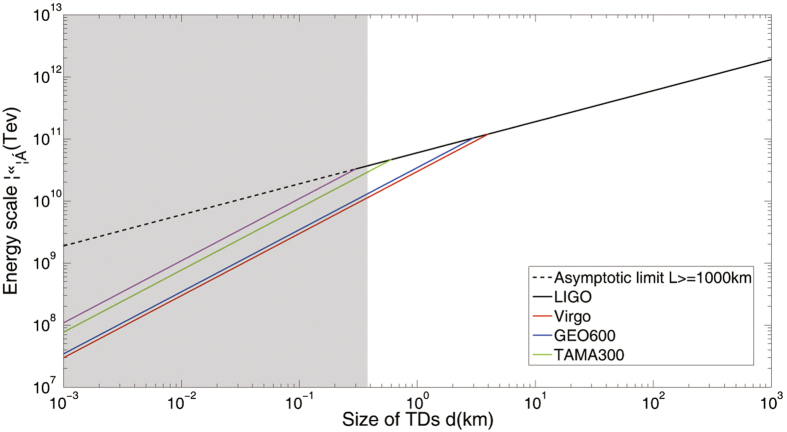
Parameter space available for the detection of the TDMs. The experimental accessible parameter space in terms of defect size(or mass) and the strength of the coupling constraints are shown in Fig. 2, fixing the characteristic time between TDMs, T 1 yr.

In consideration of the gravitational and astrophysical constraints in Eq. 15, the experimental accessible parameter space in terms of the defect size and the strength of the constraints are shown in Fig. 2, fixing the characteristic time between TDMs, T ~ 1 *yr*. Existing interferometers of various sizes are used in our scheme^22^,^23^,^24^. A comb with 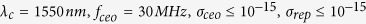 is considered in Fig. 2. The dark shaded area is the region excluded by existing laboratory and astrophysical observations^25^.

In conclusion, We demonstrated that a coherent laser ranging system, as a particularly sensitive probe, offers a realistic opportunity for detecting the topological defect form of dark matter. Our proposed experiments require either minor or no modifications of existing apparatus. In consideration of the various experimental and theoretical constraints, we demonstrate our scheme can probe parts of the important and unconstrained space of physical parameters where such defects can contribute significantly to dark matter and dark energy. Furthermore, coherent dual-comb interferometry could measure the absolute optical path length accurately, which gives our scheme a significant advantage over the cw-laser interferometer method for reducing systematic misjudgement.

## Method

**Figure 3:**
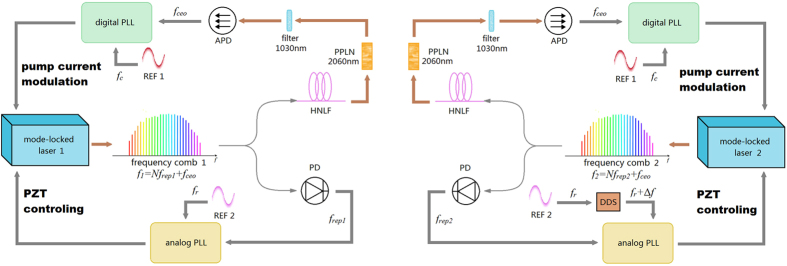
Experimental setup of the coherent dual-comb locking system. One beam of the frequency comb is used to extract and lock *f*_*ceo*_ through the f-2f self-reference method. High Nonlinear Fibre (HNLF) and a periodically poled lithium niobate (PPLN) is used to double the 2060 nm combs to 1030 nm. The other beam of frequency comb is detected by a photodetector to obtain *f*_*rep*_ and feed back to an analogue phase lock loop to control the piezoelectric transducer (PZT) in the laser for locking *f*_*rep*_. DDS is used to generate the frequency difference between two combs’ repetition rate.

Er-droped mode-locked laser can provide 1550 nm frequency comb. To realise two stable and accurate frequency combs, the carrier-envelop frequency (*f*_*ceo*_) and the repetition frequency (*f*_*rep*_) must be locked, as shown in Fig. 3. The frequency can be expressed as


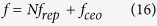


The f-2f self-reference is a commonly used method to extract (*f*_*ceo*_). One beam of the frequency comb is used to spread the spectrum to an Optical Octave from 1030 nm to 2060 nm using a High Nonlinear Fibre (HNLF), periodically poled lithium niobate (PPLN) is used to double the 2060 nm combs to 1030 nm. These processes can be expressed as


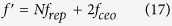


Next, the frequency comb is filtered. 1030 nm *f* and 1030 nm *f* ′ can produce a beat spectrum *f*_*ceo*_. This beat frequency comb is feed back to a digital phase lock loop to modulate the pump current of the laser. The other beam of the frequency comb is detected by a photodetector to obtain *f*_*rep*_. This photodetector signal is feed back to an analogue phase lock loop to control the piezoelectric transducer (PZT) in the laser. Using thses signals, the *f*_*ceo*_ and the *f*_*rep*_ can be locked. The *f*_*ceo*_ of frequency combs 1 and 2 are both locked to ref. 1 (*f*_c_ = 30 *MHz*). The *f*_*rep*_ of frequency comb 1 is locked to ref. 2 (*f*_*r*_ = 100 *MHz*). The *f*_*rep*_ of frequency comb 2 is locked to *f*_*r*_ + Δ*f*, which is synthesised by DDS, and the reference of DDS is *f*_*r*_.

## Additional Information

**How to cite this article**: Yang, W. *et al*. Detecting Topological Defect Dark Matter Using Coherent Laser Ranging System. *Sci. Rep.* **6**, 29519; doi: 10.1038/srep29519 (2016).
